# Interferon-Lambda Intranasal Protection and Differential Sex Pathology in a Murine Model of SARS-CoV-2 Infection

**DOI:** 10.1128/mBio.02756-21

**Published:** 2021-11-02

**Authors:** Sook-Young Sohn, Janet Hearing, JoAnn Mugavero, Varvara Kirillov, Elena Gorbunova, Luke Helminiak, Smruti Mishra, Erich Mackow, Patrick Hearing, Nancy C. Reich, Hwan Keun Kim

**Affiliations:** a Department of Microbiology and Immunology, Stony Brook University, Stony Brook, New York, USA; Duke University Medical Center

**Keywords:** COVID-19, SARS-CoV-2, antiviral, interferon, lung infection, murine model

## Abstract

Outbreaks of emerging viral pathogens like severe acute respiratory syndrome coronavirus 2 (SARS-CoV-2) are a major medical challenge. There is a pressing need for antivirals that can be rapidly deployed to curb infection and dissemination. We determined the efficacy of interferon lambda-1 (IFN-λ) as a broad-spectrum antiviral agent to inhibit SARS-CoV-2 infection and reduce pathology in a mouse model of disease. IFN-λ significantly limited SARS-CoV-2 production in primary human bronchial epithelial cells in culture. Pretreatment of human lung cells with IFN-λ completely blocked infectious virus production, and treatment with IFN-λ at the time of infection inhibited virus production more than 10-fold. To interrogate the protective effects of IFN-λ in response to SARS-CoV-2 infection, transgenic mice expressing the human angiotensin-converting enzyme 2 (ACE-2) were tested. One dose of IFN-λ administered intranasally was found to reduce animal morbidity and mortality. Our study with SARS-CoV-2 also revealed a sex differential in disease outcome. Male mice had higher mortality, reflecting the more severe symptoms and mortality found in male patients infected with SARS-CoV-2. The results indicate that IFN-λ potentially can treat early stages of SARS-CoV-2 infection and decrease pathology, and this murine model can be used to investigate the sex differential documented in COVID-19.

## INTRODUCTION

The ongoing severe acute respiratory syndrome coronavirus-2 (SARS-CoV-2) pandemic and the prior emergence of SARS-CoV in 2002 and the Middle East respiratory syndrome coronavirus (MERS-CoV) in 2012 have underscored the urgent need for effective antiviral agents that can be deployed rapidly and can combat a diversity of respiratory viral pathogens. The development of vaccines for SARS-CoV-2 has been the defining factor in controlling the coronavirus disease 2019 (COVID-19) pandemic. However, specific vaccines take many months to generate and do not address viral variants that escape vaccine immunity or the emergence of distinct pathogenic viruses.

Interferons (IFNs) are natural antiviral cytokines and are essential for an effective early defense response. Pathogen recognition receptors such as Toll-like receptors and retinoic acid-inducible gene I-like receptors are activated in response to virus and initiate signaling cascades to induce gene expression of type I IFNs (alpha and beta) and type III IFNs (lambda) (1, 2). Type I and III IFNs are secreted from infected cells and bind distinct cell surface receptors. Receptor binding stimulates the phosphorylation and activation of signal transducers and activators of transcription (STATs) that induce IFN-stimulated genes (ISGs), the main effectors of antiviral protection (3, 4). SARS-CoV-2 has evolved multiple strategies to block the induction and action of IFNs (5–8). The ability of SARS-CoV-2 to antagonize IFN innate immunity is coincident with insufficient levels of IFN found in patients early during COVID-19 (9–11). The critical role of IFN for a successful response to SARS-CoV-2 is evident in severe COVID-19 patients who produce autoantibodies to IFN and in patients with life-threatening disease who have genetic deficiencies in IFN immunity (12, 13).

Although SARS-CoV-2 can suppress the IFN response, exogenous IFN treatment has been shown to inhibit viral production in cultured lung cells (14, 15). This has led to the proposition that the administration of IFN can protect against SARS-CoV-2 infection and lessen the severity of COVID-19. This is supported by an early exploratory study with type I IFN showing a reduced duration of viral infection in the respiratory tract (16). Type I IFN receptors are expressed on most cells of the body, including immune cells. For this reason, type I IFN stimulation can trigger systemic and local inflammation by immune cell activation and recruitment to the site of infection. High levels of type I IFN can contribute to the damaging cytokine storm evident in the severe respiratory inflammation caused by SARS-CoV-2 infection. In contrast, type III IFNs (IFN-λ) bind to a different receptor whose expression is limited primarily to epithelial cells and therefore is not associated with inflammation. The potential benefit of IFN-λ as an antiviral treatment for SARS-CoV-2 infection has been proposed, and clinical trials are ongoing with subcutaneous administration of pegylated-IFN-λ (17).

We used an experimental mouse model to investigate the impact of intranasal administration of IFN-λ on SARS-CoV-2 infection and disease outcome. Since SARS-CoV-2 binds specifically to the human angiotensin-converting enzyme 2 (ACE-2) for entry, transgenic mice were used that express human ACE2 in epithelial cells of the respiratory tract. We provide evidence that intranasal administration of IFN-λ reduces animal morbidity and mortality in response to SARS-CoV-2 at a viral dose close to the estimated infectious dose in patient populations (18). These results indicate that IFN-λ could be an effective preventive or therapeutic antiviral agent to treat human SARS-CoV-2 infection. This preclinical model of infection also revealed a sex differential in disease outcome. Male mice had a higher mortality rate in response to SARS-CoV-2 than female mice. This finding reflects the more severe illness and mortality that have been well documented in male versus female COVID-19 patients worldwide (19, 20) (https://globalhealth5050.org).

## RESULTS

### Interferon-lambda inhibits infection of human bronchial epithelial cells by SARS-CoV-2.

**FIG 1:**
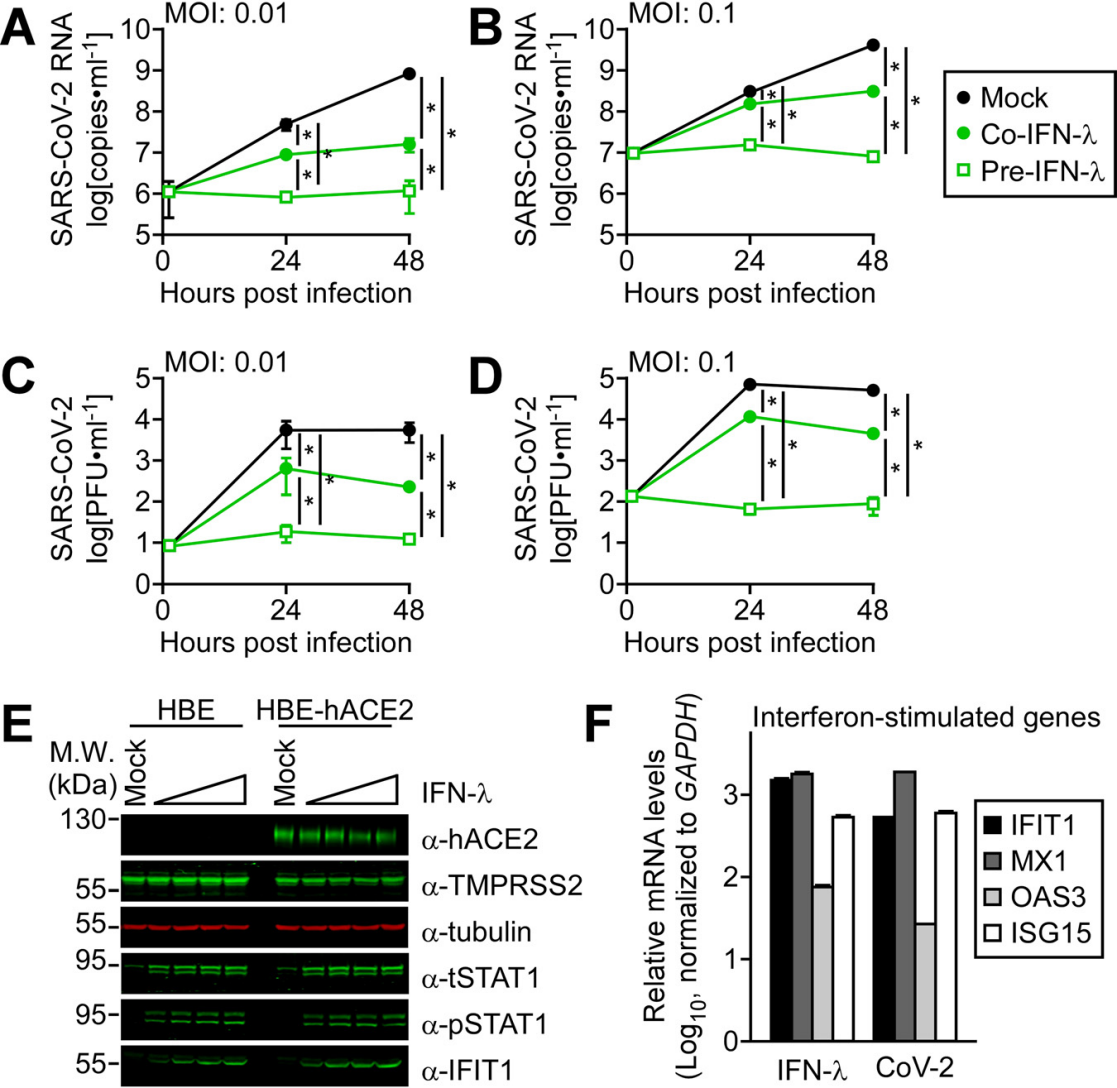
IFN-λ inhibited SARS-CoV-2 replication in cultured human lung cells. Human bronchial epithelial cells expressing hACE2 (HBE-hACE2) were either mock treated (black-filled circles), pretreated for 24 h with IFN-λ (pre-IFN-λ) (green open squares), or treated with IFN-λ at the same time of infection (co-IFN-λ) (green-filled circles) with SARS-CoV-2. Culture medium was harvested at 0, 24, and 48 h postinfection, and viral RNA in culture medium was measured by RT-qPCR following infection at MOI of 0.01 PFU/cell (A) or MOI of 0.1 PFU/cell (B). Mature virus released into the culture media was quantified by plaque assay following infection at MOI of 0.01 PFU/cell (C) or MOI of 0.1 PFU/cell (D). Three independent experiments were performed with technical duplicates. *, *P* < 0.001 (two-way ANOVA). (E) Representative Western blots are shown of proteins expressed in the HBE and HBE-hACE2 cells following treatment with increasing amounts of IFN-λ (200 to 2,000 units/ml) for 24 h. (F) RT-qPCR measurements are shown for ISG mRNAs expressed in HBE-hACE2 cells relative to untreated controls in response to IFN-λ treatment (1,000 units/ml) or relative to mock treatment in response to a SARS-CoV-2 infection for 24 h.

Coronaviruses are positive-sense RNA viruses with fast replication rates, producing virions within hours of infection. The innate immune response is therefore critical for host defense, but SARS-CoV-2 has evolved strategies to block the antiviral IFN responses. To determine the potential protective effects of IFN-λ administration, we evaluated its effects on virus production in cultured human lung cells. Infection is dependent on the presence of viral receptors and coreceptors, and the number of ACE2-expressing cells is greatest in the nasal epithelium and lower in the bronchi (21). We found that human bronchial epithelial cells immortalized with hTERT and CDK4 (HBE3-KT) were refractory to infection and expressed undetectable levels of hACE2, but they did express the TMPRSS2 cell surface protease that activates the SARS-CoV-2 spike glycoprotein. We therefore generated derivatives expressing hACE2 designated HBE-hACE2. The HBE-hACE2 cells were permissive for infection even at low multiplicities of infection (Fig. 1A to D). To investigate the effects of exogenous IFN-λ on infection by SARS-CoV-2, HBE-hACE2 cells were either pretreated with IFN-λ 24 h prior to SARS-CoV-2 infection or treated with IFN-λ at the same time of infection with a multiplicity of infection (MOI) of 0.1 or 0.01 PFU/cell. Viral RNA released in the culture media was measured by reverse transcriptase quantitative PCR (RT-qPCR) (Fig. 1A and B), and mature virus was measured by plaque assay (Fig. 1C and D). Results showed that addition of IFN-λ to the cultures during infection reduced the amount of viral RNA and virus produced by the HBE-hACE2 cells. Pretreatment with IFN-λ completely blocked the release of viral RNA and infectious virus from infected cells. Western blotting of proteins expressed in HBE and HBE-hACE2 cells showed that they responded to IFN-λ with tyrosine phosphorylation of the STAT1 transcription factor, increased steady-state levels of STAT1, and synthesis of the ISG protein IFIT1 (Fig. 1E). Analyses of RNA expression in the bronchial epithelial cells following treatment with IFN-λ showed an increase in ISG mRNA levels of IFIT1, MX1, OAS3, and ISG15 (Fig. 1F). ISG mRNA levels also increased with SARS-CoV-2 later in infection (24 h postinfection [hpi]). SARS-CoV-2 has been shown to suppresses ISG mRNA expression early in infection, and later in infection, there is an increase in ISG mRNA levels (5). The delayed ISG expression is proposed to provide a time frame for the virus to replicate unencumbered.

### Sex as a determinant of disease outcome following SARS-CoV-2 infection.

**FIG 2:**
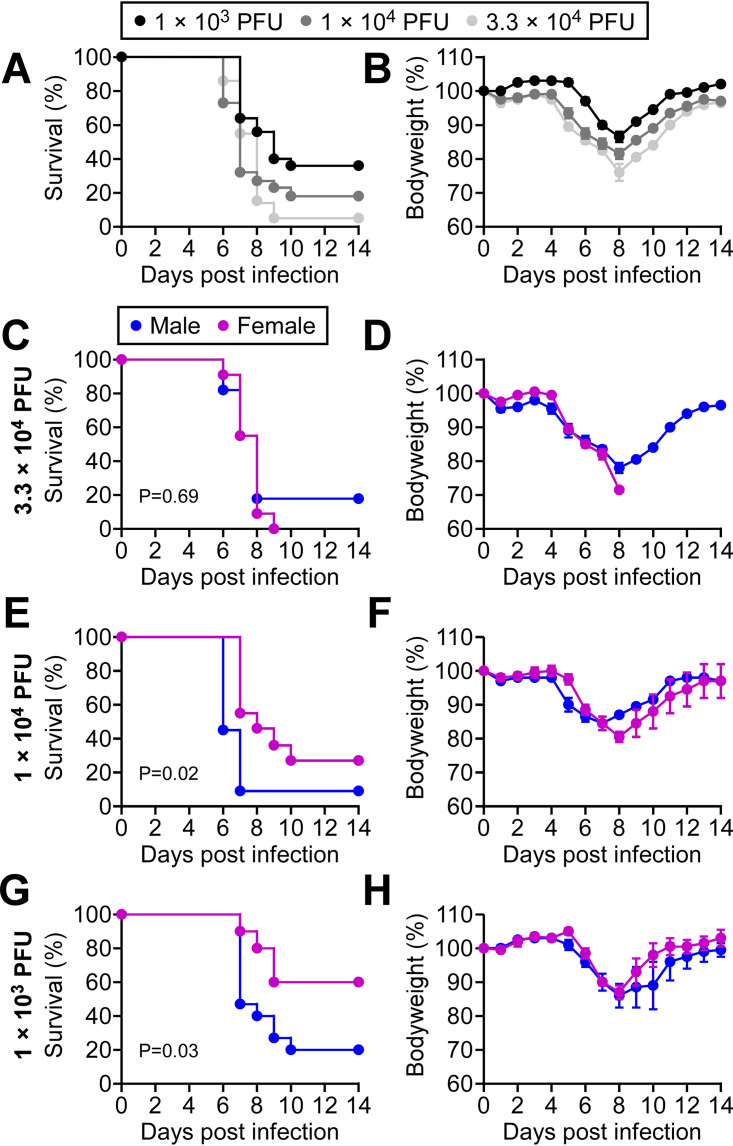
Male sex is a risk factor for death in a preclinical model of SARS-CoV-2 infection. Male and female K18-hACE2 transgenic mice were infected intranasally with three different doses of SARS-CoV-2. (A) Kaplan-Meier survival curves are presented from pooled data of three independent experiments in panels C, E, and G. (B) Averaged body weight changes are presented for each viral dose of mice in A. (C and D) Data for survival and body weight changes for 11 male and 12 female mice infected with 3.3 × 10^4^ PFU of SARS-CoV-2. Data are stratified for male sex (blue line) and female sex (pink line). (E and F) Data for survival and body weight changes for 11 male and 10 female mice infected with 1.0 × 10^4^ PFU of SARS-CoV-2. Data are stratified for male sex (blue line) and female sex (pink line) (G and H) Data for survival and body weight changes for 15 male and 10 female mice infected with 1.0 × 10^3^ PFU of SARS-CoV-2. Data are stratified for male sex (blue line) and female sex (pink line). Results represent pooled data from three independent experiments. *P* values are provided in the figure.

To interrogate COVID-19 pathology, we used a preclinical mouse model of disease and SARS-CoV-2 intranasal infection. Transgenic mice expressing human ACE2 in epithelial cells of the respiratory tract and other tissues (K18-hACE2) (22) were infected with increasing doses of SARS-CoV-2 from 1 × 10^3^ PFU to 3.3 × 10^4^ PFU (Fig. 2A). Survival decreased with a higher infectious dose; however, the animals that succumbed to disease all expired between 6 and 10 days following infection regardless of the dose. This time frame was accompanied by body weight reduction in response to infection in both survivors and nonsurvivors (Fig. 2B). We then evaluated whether there was a sex bias associated with a greater risk of death. It has been clearly documented worldwide that the severity and mortality due to COVID-19 are higher in human male patients than female patients (19, 23, 24). We found a sex differential was evident with lower viral doses in the mouse model. At the higher inoculum of 3.3 × 10^4^ PFU, male and female mice succumbed to infection with similar kinetics and outcome (Fig. 2C and D). However, at 1 × 10^4^ PFU and 1 × 10^3^ PFU, a difference between the male and female response to infection was evident (Fig. 2E and G). Males had a significant increase in mortality compared to females. These findings indicate that male susceptibility is a physiological difference independent of lifestyle or comorbidity.

### Interferon lambda administration promotes survival in a mouse model of SARS-CoV-2 infection.

**FIG 3:**
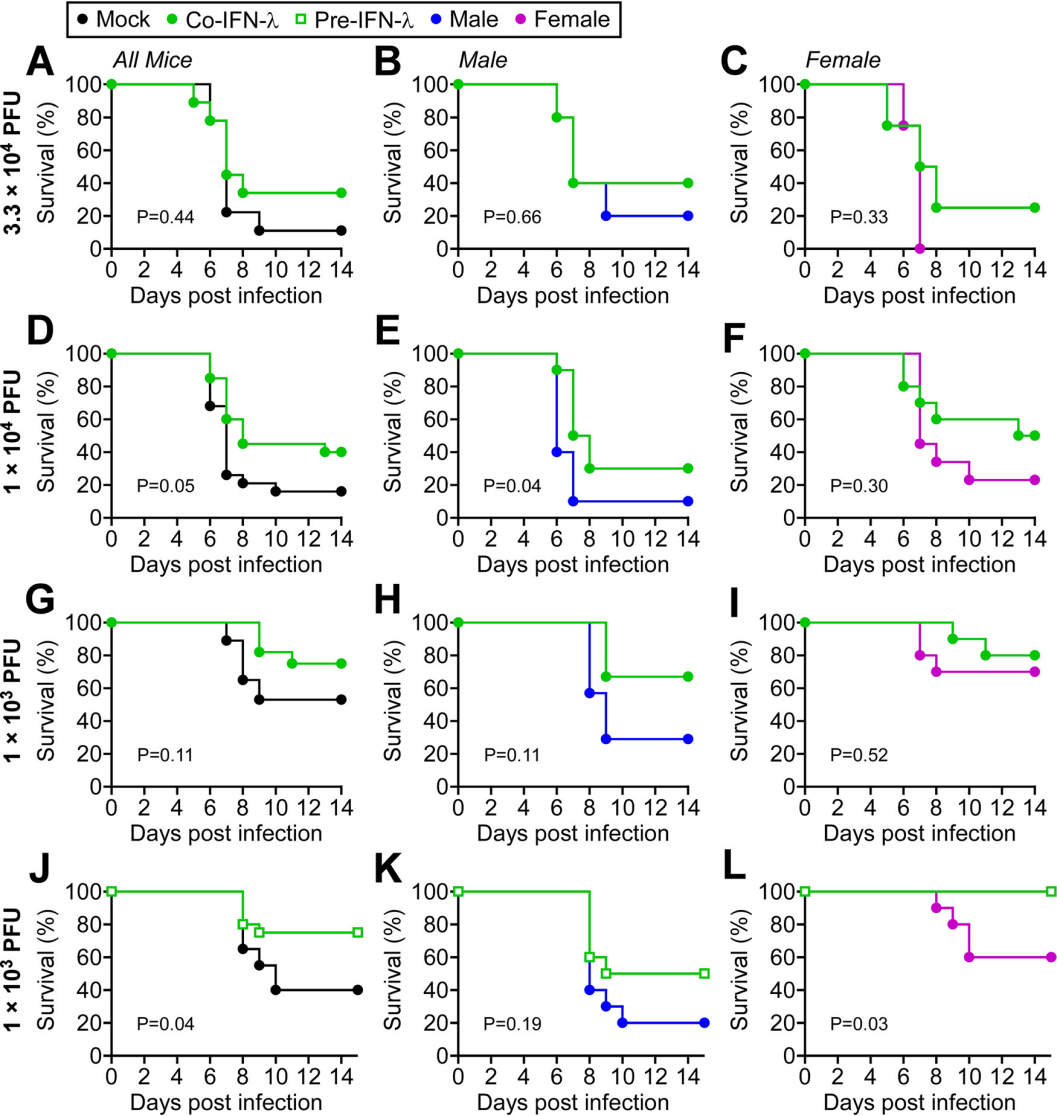
IFN-λ administration reduced mortality in a mouse model of SARS-CoV-2 infection. Male and female K18-hACE2 transgenic mice were infected with the specified dose of SARS-CoV-2, and survival is displayed as Kaplan-Meier curves. The left panels for each set of infections represent both male and female mice, and the right panels are stratified for male and female mice. (A to C) Data for survival of mice infected with 3.3 × 10^4^ PFU virus (5 male mice, 5 female mice; black closed circle) and mice treated with IFN-λ at the time of infection (5 male, 4 female; green closed circle). (D to F) Data for survival of mice infected with 1.0 × 10^4^ PFU virus (10 male, 9 female; black closed circle) and mice treated with IFN-λ at the time of infection (10 male, 10 female; green closed circle). (G to I) Data for survival of mice infected with 1.0 × 10^3^ PFU virus (7 male, 10 female; black closed circle) and mice treated with IFN-λ at the time of infection (6 male, 10 female; green closed circle). (J to L) Data for survival of mice infected with 1.0 × 10^3^ PFU virus (10 male, 10 female; black closed circles) and mice pretreated with IFN-λ for 24 h prior to infection (10 male, 10 female; green open squares). Results represent pooled data from two independent experiments. *P* values are provided in the figure.

Since IFN-λ inhibited SARS-CoV-2 infection of cultured human bronchial epithelial cells, we examined the protective effects of IFN-λ in the K18-hACE2 preclinical mouse model. Kaplan-Meier curves are shown representing the results of infection with three different doses of SARS-CoV-2 (3.3 × 10^4^ PFU, 1 × 10^4^ PFU, and 1 × 10^3^ PFU) either without administration of IFN-λ or with intranasal administration of IFN-λ at the time of infection (closed green circles) (Fig. 3A, D, and G). Corresponding body weight changes are presented in Fig. S1 in the supplemental material. Results indicate increased survival of SARS-CoV-2 infection with IFN-λ administration at lower doses of virus. Although IFN-λ afforded protection from lower inoculums of SARS-CoV-2, stratification of the results for male and female mice did not show a sex difference in IFN-λ protection (Fig. 3B, C, E, F, H, and I). The expression of ISG mRNAs in lung homogenates of mice treated with IFN-λ was similar in males and females 24 h following administration (Fig. S2). Changes in ISG expression were not detectable in whole-lung homogenates with this SARS-CoV-2 low inoculum of 1 × 10^3^ PFU (Fig. S2).

10.1128/mBio.02756-21.3 FIG S1 Body weight changes of infected mice. Body weights of SARS-CoV-2-infected mice corresponding to the results in Fig. 3. The averages of bodyweights are shown for infected mice without IFN treatment (mock) (black-filled circles), with IFN-λ treatment at the time of infection (green-filled circles), or with IFN-λ treatment 24 h prior to infection (green open squares). The specific viral doses and body weights are presented for all mice (left panels) and are stratified into survivors and nonsurvivors. Ten mice were infected with 3.3 × 10^4^ PFU virus, and 9 mice were cotreated with IFN-λ. Nineteen mice were infected with 1.0 × 10^4^ PFU virus, and 20 mice were cotreated with IFN-λ. Seventeen mice were infected with 1.0 × 10^3^ PFU virus, and 16 mice were cotreated with IFN-λ. Twenty mice were pretreated with IFN-λ and infected with 1.0 × 10^3^ PFU virus. Download FIG S1, PDF file, 2.6 MB. Copyright © 2021 Sohn et al. 2021 Sohn et al. https://creativecommons.org/licenses/by/4.0/ This content is distributed under the terms of the Creative Commons Attribution 4.0 International license.

10.1128/mBio.02756-21.4 FIG S2 ISG mRNA expression in the lungs of mice. Mice were treated with control PBS, treated with IFN-λ (2 μg), or infected with SARS-CoV-2 (1 × 10^3^ PFU) for 24 h, and lungs were harvested. Lung homogenates were prepared for RT-qPCR, and the levels of specific ISG mRNAs were measured with primers shown in Table S1. mRNA levels are shown for IFN-λ treatment or SARS-CoV-2 infection relative to the PBS control. Three mice were used for each treatment, and the mean ± SEM is presented. Download FIG S2, PDF file, 0.5 MB. Copyright © 2021 Sohn et al. 2021 Sohn et al. https://creativecommons.org/licenses/by/4.0/ This content is distributed under the terms of the Creative Commons Attribution 4.0 International license.

10.1128/mBio.02756-21.1 TABLE S1 Sequence of oligonucleotide primers used in the study. Download Table S1, DOCX file, 0.01 MB. Copyright © 2021 Sohn et al. 2021 Sohn et al. https://creativecommons.org/licenses/by/4.0/ This content is distributed under the terms of the Creative Commons Attribution 4.0 International license.

SARS-CoV-2 has developed multiple strategies to evade the IFN defense response (5–8). For this reason, administration of IFN-λ prior to infection is predicted to provide the most significant protection. To address this possibility, mice were administered IFN-λ intranasally 24 h before infection with 1 × 10^3^ PFU SARS-CoV-2, and disease progression was measured (Fig. 3J) (open green squares). IFN-λ administration had a clear preventative effect on mortality. Stratifying the IFN-λ-treated animals by sex showed that all deaths occurred in male mice (Fig. 3L). Together, the results indicate that intranasal IFN-λ administration is an effective preventative therapeutic.

### Viral clearance by IFN-λ administration in a SARS-CoV-2 mouse model of infection.

**FIG 4:**
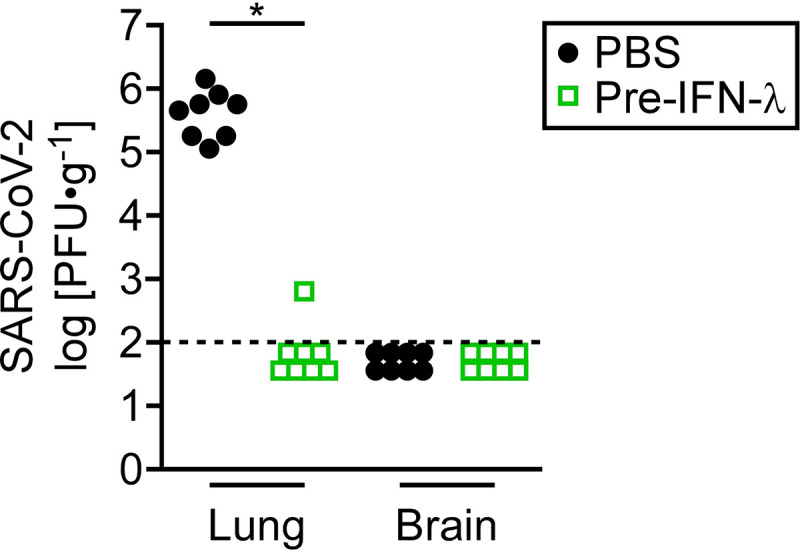
IFN-λ acts to inhibit SARS-CoV-2 replication in the lung. Male and female K18-hACE2 transgenic mice were either mock treated (black closed circles) or pretreated with intranasal IFN-λ for 24 h (green open squares) prior to infection with 1 × 10^3^ PFU SARS-CoV-2. Viral titers were measured by plaque assay in lungs and brains harvested from eight mice 2 days postinfection. The dotted horizontal line indicates the limit of detection. Circles and boxes represent individual mice. Results represent pooled data from two independent experiments. *, *P* = 0.0002 (Mann-Whitney).

To determine if the antiviral effect of IFN-λ is the primary mechanism by which IFN-λ protects animals from SARS-CoV-2 pathology, we measured virus production in IFN-λ-treated and untreated infected mice (Fig. 4). Mice were either infected intranasally with 1 × 10^3^ PFU SARS-CoV-2 or were pretreated with IFN-λ 24 h prior to the infection. The lungs and brains of infected mice were harvested 2 days postinfection, and infectious virus was measured in organ homogenates by plaque assays. There was no evidence of virus in the brains of infected mice at the limit of detection, 120 PFU/g tissue. However, the lungs from eight mice infected with virus produced titers between 1.1 × 10^5^ and 1.5 × 10^6^ PFU/g tissue. In stark contrast, seven mice that received IFN-λ before infection had no detectable virus in the lungs, and one animal had only 630 PFU/g tissue. These results indicate that the primary mode of action of IFN-λ in promoting animal survival is suppression of virus production.

### Histology reflected increased pathology in SARS-CoV-2-infected male mice.

**FIG 5:**
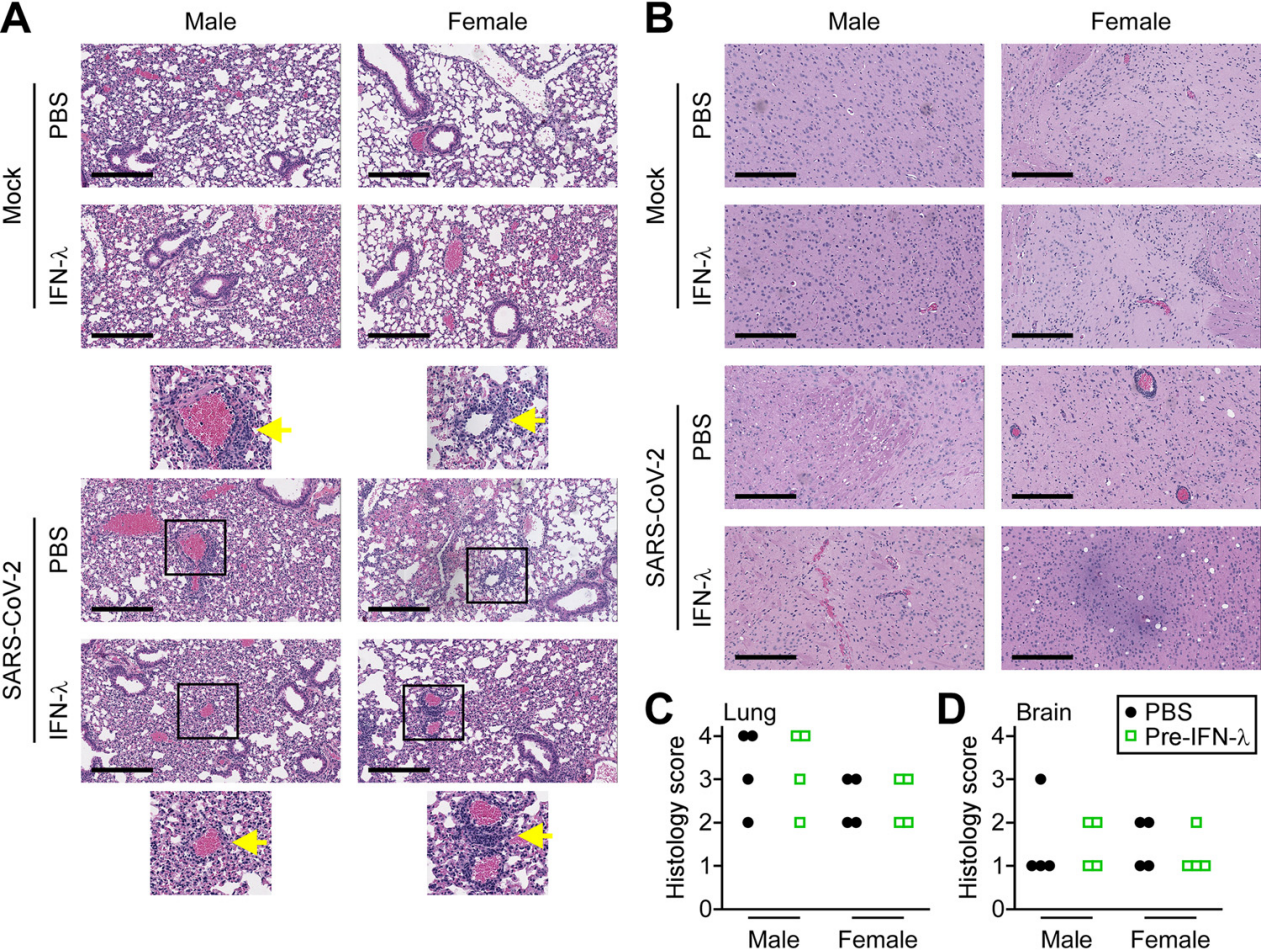
Lung and brain histology following SARS-CoV-2 infection. (A) Representative hematoxylin and eosin (H&E) staining of mouse lung tissue sections. Top panels show images of lungs from uninfected mice (mock) treated with PBS or IFN-λ for 5 days. Bottom panels show representative lung sections from mice that were pretreated with PBS or IFN-λ and infected with 1 × 10^3^ PFU SARS-CoV-2 for 5 days. Scale bar, 300 μm. Magnified images are shown with arrows identifying perivascular immune cell infiltration. (B) H&E-stained sections of brain tissue from mice treated as in panel A. Quantitative histology scoring for lungs (C) and brains (D) of male and female mice is described in Table S2 in the supplemental material.

The pathology in mouse brains and lungs caused by virus infection was assessed by histology. Histology scoring parameters are provided in Table S2. Lungs and brains of mice were harvested 5 days following intranasal infection and evaluated for inflammation and hemorrhage (Fig. 5). Mock phosphate-buffered saline (PBS)-treated mice showed normal lung architecture with thin alveolar walls and clear air space. Following SARS-CoV-2 infection, a greater number of male mice showed moderate to severe bronchitis and perivascular lung inflammatory cells in comparison to less severe pathology in infected female mice. The difference is consistent with the sex differential in survival data (Fig. 2 and 3). Administration of IFN-λ alone had a minor inflammatory effect in the lungs. There were no obvious changes in the brains of male or female mice following infection with SARS-CoV-2 (Fig. 5B and D).

10.1128/mBio.02756-21.2 TABLE S2 Criteria used to score histopathology of mouse lungs in Fig. 5. Download Table S2, DOCX file, 0.01 MB. Copyright © 2021 Sohn et al. 2021 Sohn et al. https://creativecommons.org/licenses/by/4.0/ This content is distributed under the terms of the Creative Commons Attribution 4.0 International license.

## DISCUSSION

SARS-CoV-2 is a positive-strand RNA virus with rapid replication kinetics and an estimated doubling time of 6 h (25). Therefore, an effective defense response is dependent on the swift action of innate immunity. Both type I IFN (IFNs-α/β) and type III IFN (IFN-λ) are critical components of the first line of innate defense. They bind distinct cell surface receptors to induce a similar set of IFN-stimulated genes (ISGs) that function in antiviral protection. IFNs-α/β receptors are expressed on all somatic cells, including immune cells; however, IFN-λ receptors are limited principally to epithelial cells (26). IFN-λ does not activate macrophages, T cells, or other cells of the immune system responsible for the cytokine storm and inflammation that are coincident with SARS-CoV-2 acute respiratory syndrome. Consequently, IFN-λ plays a protective role, primarily at mucosal sites such as the respiratory tract. For this reason, we investigated the potential therapeutic value of intranasal administration of IFN-λ as an antiviral in response to SARS-CoV-2 infection. The effects of IFN-λ on SARS-CoV-2 infection were first evaluated in cultured human lung cells. Our results and those of others demonstrated that IFN-λ inhibits SARS-CoV-2 production in cultured human lung cells (Fig. 1) (14, 15). IFN-λ addition at the time of infection reduced viral titers, and pretreatment of cells with IFN-λ completely blocked SARS-CoV-2 from establishing an infection. This differential effect is not unexpected since hours are needed for host cells to express antiviral ISG proteins in response to IFN-λ.

The human ACE2 metalloprotease has been identified as the primary functional receptor for SARS-CoV and for SARS-CoV-2 (27, 28). Originally, ACE2 was reported to be an ISG, raising concerns for the efficacy of IFN-based therapies for COVID-19 (29). However, it was later shown that IFN only induces expression of a truncated product of ACE2 that is not able to bind SARS-CoV-2 spike protein (30–32). Because the SARS-CoV-2 spike protein binds poorly to murine ACE2, we tested the prophylactic and therapeutic effects of IFN-λ in infected mice expressing the human ACE2 transgene driven by the cytokeratin-18 promoter (K18-hACE2) (33, 34).

Our studies with intranasal infection with a low dose of SARS-CoV-2 revealed a sex difference in disease outcome (Fig. 2). The precise infectious dose of SARS-CoV-2 during human respiratory transmission remains to be determined, but a computational estimate is approximately 300 virus particles (18). In our study, a high viral dose (3.3 × 10^4^ PFU) produced a rapidly fatal disease in both male and female mice. However, as the inoculum was reduced to 1 × 10^3^ PFU, male mice were found to have a higher mortality rate. This sex differential is reflective of the more severe symptoms and mortality that are well documented in male patients that suffer from COVID-19 (19, 23, 35).

Since the hACE2 gene is located on the X chromosome, it has been speculated that variants of hACE2 could be more detrimental in men and contribute to the sex differential. However, hACE2 in the transgenic mice is independent of X chromosome location, encodes the same gene in male and female mice, and is regulated by the same promoter. It has also been proposed that contributing factors of smoking or comorbid conditions of diabetes or heart disease may impact the sex differential, but these are not factors with the transgenic mice. A known difference between males and females is the increased responsiveness of the RNA-sensing Toll-like receptor 7 in females that leads to increased IFN expression (36, 37). Infection of a low number of lung cells with 1 × 10^3^ PFU SARS-CoV-2 did not detect changes in ISG mRNA expression in lysates of male or female lungs (Fig. S2 in the supplemental material). Understanding the sex bias in response to SARS-CoV-2 infection is critical in considering therapeutic intervention and prediction of patient outcome. The transgenic model system will be used in the future to investigate the mechanisms that lead to worsened outcome in males following SARS-CoV-2 infection.

IFNs have been approved clinically for the treatment of several cancers, autoimmune diseases, and viral infections (38). The potential therapeutic value of IFN-λ for COVID-19 has been examined in clinical trials with a single subcutaneous injection of a pegylated form of IFN-λ (pegIFN-λ) either 3 days following diagnosis of mild to moderate COVID-19 (39) or within 7 days of symptoms or diagnosis (40). The former trial found no harmful effects of IFN-λ but no improvement in symptoms, whereas the latter trial found an improvement in viral clearance and respiratory symptoms. Experiments with a mouse-adapted SARS-CoV-2 indicated subcutaneous injection of pegIFN-λ reduced mortality in mice, although the animals were only evaluated for 5 days (41). Because SARS-CoV-2 infection commonly leads to an acute respiratory syndrome, intranasal administration or inhalation may be more therapeutic than subcutaneous injections. For this reason, we evaluated the protective effects of intranasal administration of unmodified IFN-λ in the K18-hACE2 transgenic mice with different doses of SARS-CoV-2. Overall, IFN-λ treatment at the time of viral infection was found to maintain body weight of survivors and reduce mortality (Fig. 3; Fig. S1). IFN-λ had even greater protection if administered 1 day prior to SARS-CoV-2 infection. In fact, all female mice survived infection with 1 × 10^3^ PFU with IFN-λ pretreatment. The preventative value of IFN-λ is not unexpected since the antiviral ISGs are expressed before the SARS-CoV-2 products can inhibit IFN signaling and ISG expression (5–8).

To investigate the mechanism by which IFN-λ promotes survival following SARS-CoV-2 infection, we measured viral titers in lungs and brains of mice 2 days postinfection. The lungs of infected mice showed substantial viral production, whereas there was nearly undetectable virus in mice that received intranasal administration of IFN-λ prior to infection (Fig. 4). The results indicate that the protective effects of IFN-λ against SARS-CoV-2 infection in this model are mediated, in large part, by the ability of IFN-λ to block infectious virus production. However, since not all of the mice survive, it is possible that multiple doses of IFN-λ are needed to completely eliminate the virus. Alternatively, infection may trigger an irreversible inflammatory response leading to the death of a mouse.

Our preclinical model showed that administration of IFN-λ can reduce morbidity and mortality caused by SARS-CoV-2 infection and that IFN-λ can be effective as a preventative therapy. Previously, type I IFN-α was used in nasal drops of healthy medical staff in the COVID-19 epidemic area of Hubei Province in China, and the results indicated protection (42). The findings in our study indicate that intranasal administration of the less inflammatory IFN-λ can reduce replication of virus in the lungs of mice and improve survival. Our findings are particularly relevant for the use of broad-spectrum antivirals against newly emergent pathogens and for disease prevention in emergency responders and health care workers.

## MATERIALS AND METHODS

### Biocontainment.

All experiments conducted with SARS-CoV-2 were conducted either in a biosafety level 3 laboratory located in the Center for Infectious Disease (CID) or an animal biosafety level 3 laboratory located in the Laboratory of Comparative Medicine, Division of Laboratory Animal Resources. All personnel completed safety training and used personal protective equipment that included powered air-purifying respirators, Tyvek coveralls and booties, and double gloves. All protocols involving infectious virus and protocols for the inactivation of virus in samples prepared for export to a biosafety level 2 laboratory were approved by the University CID Safety Committee.

### Mouse model and infections.

This study was carried out in strict accordance with the recommendations in the Guide for the Care and Use of Laboratory Animals of the NIH. The protocol was approved by the Institutional Animal Care and Use Committee of Stony Brook University (IACUC no. 1596316). The Division of Animal Laboratory Research at Stony Brook University operates in accordance with the American Association for Laboratory Animal Science (AALAS), the American College of Laboratory Animal Medicine (ACLAM), and Animal Welfare Assurance ID D16-00006 (A3011-01) of the NIH. Mice were housed in either standard filter-topped shoebox microisolator cages or filter-ventilated cages. Rooms had 10 to 15 air changes per hour and were maintained at 70 to 72°F. Pelleted irradiated Purina mouse chow was provided *ad libitum*, and hyperfiltered water (2 μm) was provided via water bottles *ad libitum*. All mice were provided with Enviro-Dri nesting material. Mice were euthanized with 3 liter/min carbon dioxide inhalation, consistent with the recommendations of the panel on euthanasia of the American Veterinary Medical Association (AVMA guidelines) and Stony Brook University IACUC.

K18-hACE2 transgenic mice [Jackson Laboratory, B6.Cg-Tg(K18-ACE2)2Prlman/J, 9 to 10 weeks old] were anesthetized via intraperitoneal injection of 100 mg·ml^−1^ ketamine and 20 mg·ml^−1^ xylazine per kilogram of body weight. Mice were infected by intranasal inoculation of 1 × 10^3^, 1 × 10^4^, or 3.3 × 10^4^ PFU SARS-CoV-2 passage 4 stock in 20 μl PBS containing 0.1% bovine serum albumin (BSA) and monitored daily for signs of disease and weight loss. For IFN-λ pretreatment, anesthetized animals received intranasal inoculation of 2 μg IFN-λ (30,000 units) in 20 μl PBS with 0.1% BSA. For IFN-λ treatment at the time of SARS-CoV-2 infection, anesthetized animals received SARS-CoV-2 mixed with 2 μg IFN-λ (30,000 units) IFN-λ in 20 μl PBS with 0.1% BSA. On day 2 following infection, mice were euthanized, lungs and brains were removed and homogenized in 2 ml Dulbecco’s modified Eagle medium (DMEM) with 10% FBS, and the viral titers in tissues were determined by plaque assay. For histopathology analysis, lungs and brains were harvested on day 5 postinfection and fixed in 10% formalin for 7 days at room temperature. Tissues were embedded in paraffin, thin sectioned, stained with hematoxylin-eosin, and inspected by light microscopy.

### Cells, virus, and IFN-λ.

Human bronchial epithelial (HBE) 3-KT cells were obtained from American Type Culture Collection (ATCC) and cultured in airway epithelial cell basal medium supplemented with the bronchial epithelial cell growth kit (ATCC). HEK293-FT cells were cultured in DMEM containing 10% fetal bovine serum (FBS) and penicillin-streptomycin. Vero E6 cells were maintained in DMEM supplemented with 10% HyClone iron-supplemented bovine calf serum (Cytiva). All cells were cultured at 37°C in a humidified atmosphere with 5% CO_2_. The SARS-CoV-2 strain (SARS-CoV-2/USA-WA1/2020) was obtained from Biodefense and Emerging Infections Research Resources Repository (NR no. 52281; BEI Resources, VA, USA). Virus stocks were prepared by passage of the seed virus (passage 1 [p1]) at an MOI of 0.01 per cell, and the infected cell culture supernatant was collected at 72 hpi, clarified by centrifugation at 400 × *g* for 10 min, and then stored at −70°C. Human IFN-λ1 (IL-29) was purchased from PBL Assay Science and diluted in PBS with 0.1% BSA.

### Generation of HBE-hACE2 cells.

The human ACE2 cDNA was amplified from pcDNA3.1-hACE2-C9 (Addgene plasmid no. 1786) (27) by PCR using the primers listed in Table S1 in the supplemental material, and the SpeI/XhoI-digested fragment was inserted into corresponding sites of pLenti6/v5-D-TOPO (Life Technologies; kindly provided by Thomas Stamminger, Ulm University). HEK293-FT cells were cotransfected with pLenti6-hACE2, pLP1, pLP2, and pVSV-G using Lipofectamine 2000 (Invitrogen) according to the manufacturer’s instructions. Twenty hours later, cells were washed with PBS and incubated with fresh medium for 2 days. The supernatant was collected and added to HBEC3-KT cells with 5 μg polybrene/ml. Forty-eight hours postransduction, cells were treated with 10 μg blasticidin S/ml (Thermo Fisher), and resistant cells were evaluated.

### Plaque assay.

Vero E6 cells were seeded in 6-well cell culture plates at 4.8 × 10^5^ cells per well and incubated for 2 days to obtain confluent monolayers. Cells were infected with 0.4 ml of sample diluted in growth medium for 1 h at 37°C with occasional rocking. The virus inoculum was removed and replaced with 2.8 ml of an overlay medium consisting of DMEM, 2.5% bovine calf serum, and 0.76% gum tragacanth. Plaques were allowed to develop by incubating for 44 to 48 h, at which time the overlay medium was aspirated, and the cells were fixed and stained for 15 min with 0.5% crystal violet, 50% methanol, and 0.8% glutaraldehyde.

### Western blot analyses.

Total cell extracts were prepared in sample buffer and subjected to SDS-PAGE. Separated proteins were transferred to nitrocellulose membranes and blocked with Tris-buffered saline containing 0.1% Tween 20 (TBST) and 3% BSA. The following primary antibodies were used: ACE2 (catalog no. 10108-T24; Sino Biological; 1:2,500), TMPRSS2 (catalog no. 14437-1-AP; Proteintech; 1:2,000), STAT1 (catalog no. 9172; Cell Signaling Technology; 1:2,000), phospho-STAT1 (Y701) (catalog no. 9167; Cell Signaling Technology; 1:2,000), IFIT1 (catalog no. 23247-1-AP; Proteintech; 1:2,000), and α-tubulin (catalog no. T5182; MilliporeSigma; 1:5,000). The IRDye 800CW-conjugated goat anti-rabbit IgG (Li-COR; 1:5,000) and the IRDye 680CW-conjugated goat anti-mouse IgG (Li-COR, 1:5,000) were used as secondary antibodies. The Western blot images were acquired on an Odyssey CLx imaging system (Li-COR) and analyzed using Image Studio software (Li-COR).

### Quantification of SARS-CoV2 RNA and cellular mRNA by RT-qPCR.

The SARS-CoV2 RNA in the cell culture media was isolated using the QIAamp viral RNA minikit (Qiagen) according to the manufacturer's protocol. RNA levels were determined by RT-qPCR using the primers listed in Table S1 and a TaqPath 1-Step RT-qPCR master mix (Thermo Fisher) on a StepOne Plus real-time PCR system (Applied Biosystems). To determine the viral RNA copy numbers, a standard curve was generated by using SARS-CoV-2 nucleocapsid RNA. Briefly, a SARS-CoV2 nucleocapsid plasmid (Addgene; plasmid no. 153201) was linearized by digestion with XbaI and purified using a PCR purification kit (Qiagen). The nucleocapsid gene was *in vitro* transcribed using a MAXIscript SP6 kit (Thermo Fisher), resulting in a 1,533-base RNA fragment. DNA in the reaction mixture was removed by incubation with Turbo DNase (Thermo Fisher), and RNA was further purified using an RNeasy minikit (Qiagen).

Cell culture lysates and mouse lung and brain homogenates were prepared by disruption in 2 ml buffer RLT (Qiagen) supplemented with 1% 2-mercaptoethanol. Total RNA was purified with an RNeasy Plus minikit (Qiagen) followed by reverse transcription using EcoDry premix (TaKaRa). cDNA levels were measured by qPCR using primers listed in Table S1 and a DyNAmo HS SYBR green master mix (Thermo Fisher). The fold change in gene expression was determined by the threshold cycle (ΔΔ*CT*) method using a differential with GAPDH (glyceraldehyde-3-phosphate dehydrogenase) and comparison with the untreated control.

### Statistical analyses.

Statistical analyses were performed with GraphPad Prism 9. The proportion of surviving animals was analyzed using the two-tailed log-rank test. The organ viral burden was analyzed by the Mann-Whitney test. Two-way analysis of variance (ANOVA) with Turkey’s multiple comparisons was performed to calculate log-transformed *in vitro* data.
