# Supplementary figures and images for: Interferon-Lambda Intranasal Protection and Differential Sex Pathology in a Murine Model of SARS-CoV-2 Infection
Source: mBio. 2021 Nov 2;12(6):e02756-21. doi: 10.1128/mBio.02756-21 (PMC8561397; doi:10.1128/mBio.02756-21)

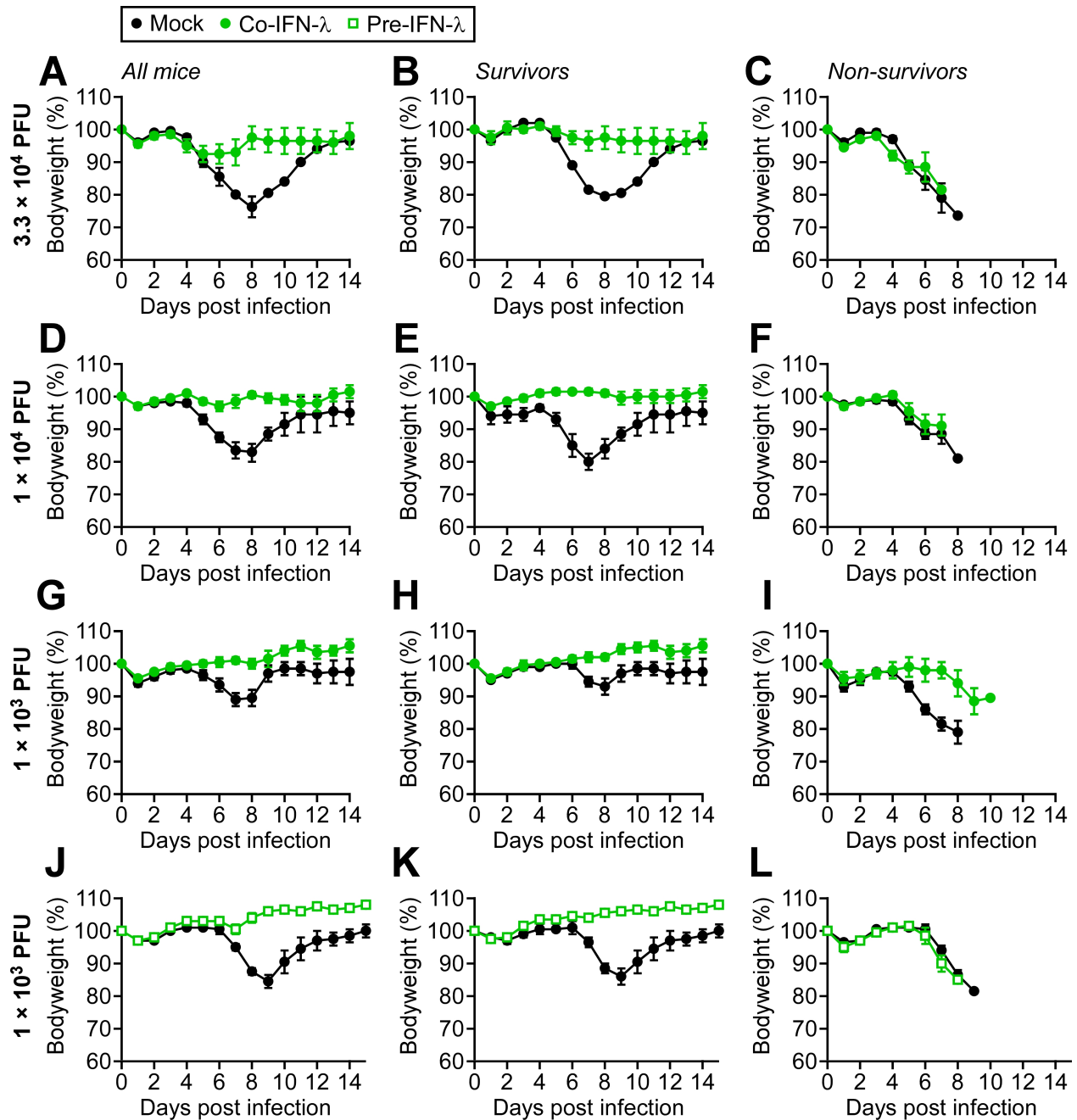

Figure S1

Supplement: FIG S1 [file mbio.02756-21-sf001.pdf]

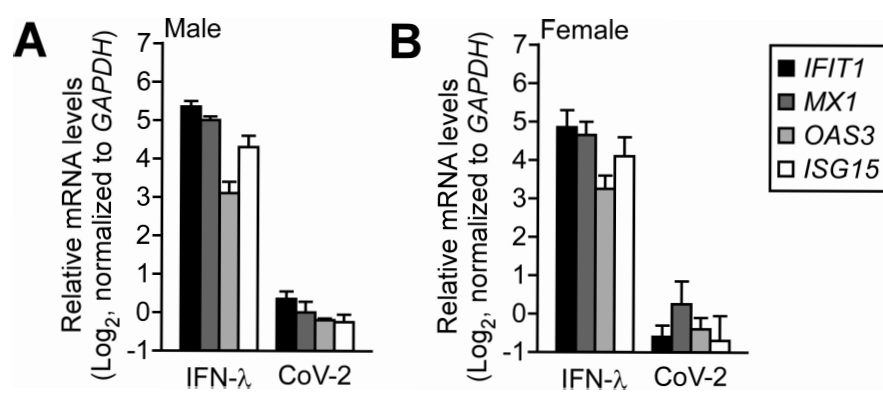

Figure S2

Supplement: FIG S2 [file mbio.02756-21-sf002.pdf]
