# Supplementary material for: Interferon-Lambda Intranasal Protection and Differential Sex Pathology in a Murine Model of SARS-CoV-2 Infection
Source: mBio. 2021 Nov 2;12(6):e02756-21. doi: 10.1128/mBio.02756-21 (PMC8561397; doi:10.1128/mBio.02756-21)
Supplement: TABLE S1 [file mbio.02756-21-st001.docx]

Table S1: Sequence of oligonucleotide primers used in the study.

| **Gene** | **Forward primer** | **Reverse primer** |
| --- | --- | --- |
| *RT-qPCR*  *Mouse* |  | |
| *GAPDH* | CCTGCACCACCAACTGCTTAG GTGGATGCAGGGATGATGTTC | |
| pan*IFNA* | CTCCTAGACTCCTTCTGCAA | AGGGGCTGTGTTTCTTCTCT |
| *IFNB* | CTGGAGCAGCTGAATGGAAAG | CTTCTCCGTCATCTCCATAGGG |
| *IFNL* | AGTGGAAGCAAAGGATTG | GAGATGAGGTGGGAACTG |
| *IFIT1* | GAGCCAGAAAACCCTGAGTACA | AGAAATAAAGTTGTCATCTAAATC |
| *MX1* | GACCATAGGGGTCTTGACCAA | AGACTTGCTCTTTCTGAAAAGCC |
| *OAS1* | ATGGAGCACGGACTCAGGA | TCACACACGACATTGACGGC |
| *ISG15* | CAATGGCCTGGGACCTAAA | CTTCTTCAGTTCTGACACCGTCAT |
| *TNFA* | CATCTTCTCAAAATTCGAGTGACAA | TGGGAGTAGACAAGGTACAACCC |
| *IL-1B* | CAACCAACAAGTGATATTCTCCATG | GATCCACACTCTCCAGCTGCA |
| *IL6* | GAGGATACCACTCCCAACAGACC | AAGTGCATCATCGTTGTTCATACA |
| *RT-qPCR Human* | | |
| *GAPDH* | ACCCAGAAGACTGTGGATGG | TTCTAGACGGCAGGTCAGGT |
| pan*IFNA* | CACACAGGCTTCCAGGCATTC | TCTTCAGCACAAAGGACTCATCTG |
| *IFNB1* | ACGCCGCATTGACCATCTATG | CGGAGGTAACCTGTAAGTCTGT |
| *IFNL1* | CGCCTTGGAAGAGTCACTCA | GAAGCCTCAGGTCCCAATTC |
| *IFIT1* | TCTCAGAGGAGCCTGGCTAA | TGCTCCAGACTATCCTTGACCT |
| *MX1* | AGCTCGGCAACAGACTCTTC | GATGATCAAAGGGATGTGGC |
| *OAS3* | CTGAAGAGCTGGACGGATGT | GGTGAGGAGCCTCGAGTAGA |
| *ISG15* | GCGAACTCATCTTTGCCAGT | AGCATCTTCACCGTCAGGTC |
| *TNFA* | GAGGCCAAGCCCTGGTATG | CGGGCCGATTGATCTCAGC |
| *IL-1B* | TACCTGTCCTGCGTGTTGAA | TCTTTGGGTAATTTTTGGGATCT |
| *IL6* | GATGAGTACAAAAGTCCTGATCCA | CTGCAGCCACTGGTTCTGT |
| *Cloning* | | |
| *ACE2* | CTAGTCACTAGTACCATGTCAAGCTCTTCCTGG | CTAGCTTCTGAGCTAAAAGGAGGTCTGAAC |
| *SARS-CoV-2* | | |
| *N1* | GACCCCAAAATCAGCGAAAT | TCTGGTTACTGCCAGTTGAATCTG |

SARS-CoV2_N1 Probe : FAM-ACC CCG CAT TAC GTT TGG TGG ACC-BHQ1
