# Supplementary material for: Interferon-Lambda Intranasal Protection and Differential Sex Pathology in a Murine Model of SARS-CoV-2 Infection
Source: mBio. 2021 Nov 2;12(6):e02756-21. doi: 10.1128/mBio.02756-21 (PMC8561397; doi:10.1128/mBio.02756-21)
Supplement: TABLE S2 [file mbio.02756-21-st002.docx]

Table S2: Criteria used to score histopathology of mouse lungs in Figure 5.

| **Scores** | **Organs affected (%)** | **Severity** |
| --- | --- | --- |
| *Lungs* |  |  |
| 0 | <10% | Within normal limits |
| 1 | <20% | Mild peribronchitis, perivascular inflammation, and immune |
|  |  | cell infiltration |
| 2 | <40% | Mild to moderate peribronchitis, perivascular inflammation, and presence of inflammatory cells |
| 3 | <60% | Moderate peribronchitis, perivascular inflammation, |
|  |  | bronchiolar epithelial necrosis, and interstitial inflammation |
| 4 | <80% | Severe bronchitis, perivascular inflammation, and endothelitis, and widespread epithelial necrosis, |
|  |  | hemorrhage, fibrin, and edema |
| *Brains* |  |  |
| 0 | <10% | Within normal limits |
| 1 | <20% | Mild inflammation, minimal necrosis, and immune cell |
|  |  | infiltration |
| 2 | <30% | Moderate inflammation and immune cell infiltration |
| 3 | <40% | Severe inflammation, ischemic lesions, and hemorrhage |
